# Fermented yellow mombin juice using *Lactobacillus acidophilus* NRRL B-4495: Chemical composition, bioactive properties and survival in simulated gastrointestinal conditions

**DOI:** 10.1371/journal.pone.0239392

**Published:** 2020-09-24

**Authors:** Ellane Sabryna Sena Ribeiro, Karla Suzanne Florentino Silva Chaves Damasceno, Livia Maria da Costa Dantas, Wendell Medeiros de Azevedo, Pedro Ivo Palacio Leite, Cristiane Fernandes de Assis, Francisco Caninde de Sousa Junior

**Affiliations:** 1 Nutrition Graduate Program, Federal University of Rio Grande do Norte, Natal, RN, Brazil; 2 Department of Nutrition, Federal University of Rio Grande do Norte, Natal-RN, Brazil; 3 Department of Pharmacy, Federal University of Rio Grande do Norte, Natal-RN, Brazil; Institute for Biological Research "S. Stanković", University of Belgrade, SERBIA

## Abstract

The purpose of the present study was to evaluate yellow mombin (*Spondias mombin* L.) juice as a vehicle for the *Lactobacillus acidophilus* NRRL B-4495 probiotic. The initial pH and fermentation temperature conditions were optimized by central composite rotational design. The beverage was evaluated for its chemical composition, bioactive properties, microbiological stability, survival in simulated gastrointestinal conditions and sensory analysis. The ideal conditions for probiotic juice production were an initial pH of 6.4 and 16 h of fermentation, with maximum viability of 12.9 ± 0.4 Log CFU/mL. The fermented juice showed a total phenolic concentration of 94.90 ± 7.12 GAE/mL and antioxidant activity, as measured by DPPH (0.31 ± 0.00 μmol TE/mL) and ABTS sequestration (2.59 ± 0.30 μmol TE/mL). Antibacterial activity could also be observed against *S*. *aureus*, *E*. *coli* and *K*. *pneumoniae*. The obtained formulation showed good microbiological stability when stored at 4ºC for 28 days. In addition, there was no significant change in viability after exposure to simulated gastrointestinal conditions. The sensory analysis showed that the probiotic beverage was not well accepted. However, the Just-About-Right (JAR) ideal scale test enabled identifying the specific attributes which need to be improved from the tasters’ point of view so that it is possible to improve product acceptance.

## Introduction

Consumers are currently more aware of the role of food in longevity, quality of life and in the prevention of chronic non-communicable diseases [1]. In this sense, the consumption of probiotic foods (defined as viable microorganisms which exhibit beneficial effects on host health when ingested in adequate amounts) has been growing in recent years [2,3].

Probiotics exert beneficial effects on host health through various mechanisms such as immune response modulation, production of organic acids and antimicrobial compounds, interaction with resident microbiota and interface with the host, thereby improving intestinal barrier integrity and enzyme production [4].

The majority of fermented beverages have been developed in dairy products. However, the growth of vegan tendencies, milk intolerance/allergy and the preference for hypocholesterolemic diets have led to the need to innovate and develop probiotic products [5–7]. Therefore, studies on the inoculation of probiotic microorganisms in fruit and vegetable juice have shown promise and have been gaining ground as an option for developing new products [8].

It is known that a food matrix used as a vehicle for probiotic microorganisms may influence their growth, viability, survival, acid and bile salt tolerance, as well as functional properties [9–11]. The most commonly used probiotic strains in food matrices belong to the *Lactobacillus* and *Bifidobacterium* genera. *L*. *acidophilus* is classified as a homofermentative bacteria which exclusively converts hexoses to acids [12]. In addition to dairy products, this species has been showing positive results when inoculated in vegetable products such as fruit juices [13,14]. Various studies have investigated the suitability of *Lactobacillus* spp. on different tropical fruit juices, e.g., sweet lemon [5], cashew apple [9], cocoa [8], passion fruit [10], cantaloupe melon [15], cupuassu [16] and guava [17] for probiotic beverages production.

Yellow mombin (*Spondias mombin* L.) is a regional 3–4 cm elliptical fruit [18]. It is rich in carbohydrates (13.9 g/100 g), proteins (1.1 g/100 g), fibers (1.9 g/100 g), pro-vitamin A (223.0 RE/100 g), β-criptoxanthin (1708.5 μg/100 g), lutein (634.0 μg/100 g), zeinoxanthin (547.5 μg/100 g), β-carotene (314.0 μg/100 g), phenolic compounds (260.2 mg GAE/100 g), magnesium (15.1 mg/100 g), phosphorous (32.8 mg/100 g), potassium (288.3 mg/100 g) and calcium (11.0 mg/100 g) [19]. This unique composition has shown antioxidant effects [19], gastroprotective and ulcer healing activities [20], attenuation of ventricular remodeling after myocardial infarction [21], and cytotoxic effects on ovarian cancer cells [22].

A few studies have evaluated the effect of adding probiotic cultures to yellow mombin ice creams [23,24], but none have evaluated its fermented juice. Therefore, the purpose of the present study was to evaluate yellow mombin juice as a vehicle for *L*. *acidophilus* NRRL B-4495 by determining its chemical composition, bioactive properties, survival in simulated gastrointestinal conditions and sensory analysis.

## Material and methods

### Yellow mombin juice

The yellow mombin (*Spondias mombin* L.) used in this study was purchased in commercial establishments in Natal, RN, Brazil. The fruits were selected and sanitized with a 1% sodium hypochlorite solution for 15 min. The fruits were then washed with running water, crushed in a processor and pressed for juice extraction. The extracted crude juice was filtered through a 48 mesh filter. The initial total soluble solids value (13.7 ºBrix) was adjusted to 8.0 ºBrix adding potable water. The juice was pasteurized (80°C for 5 min) and stored in sterile bottles at -20°C before use. The present study was conducted under the authorization from the National System for Management of Genetic Heritage and Associated Traditional Knowledge (SisGen/Brazil) no. AB1B7B2.

### Microorganism and inoculum preparation

The *Lactobacillus acidophillus* NRRL B-4495 strain was obtained from the ARS Culture Bacterial Collection (NRRL Culture Collection, U.S. Department of Agriculture, Peoria, Illinois).

In order to activate the microorganism, 8 mL of the 50% glycerol stock culture containing 9.0 Log of colony forming units per mL (Log CFU/mL) was aseptically transferred to Erlenmeyer flasks containing 100 mL of MRS (Man, Rogosa and Sharpe) broth (Becton Dickinson and Company, Sparks, MD, USA) and 10 mL of 200 mM phosphate buffer pH 6.5. This initial cultivation was performed at a temperature of 37°C for 12 h, as previously described by Fonteles et al. [15]. This suspension was used as the inoculum for the fermentation process.

### Optimization of initial pH and fermentation temperature conditions using experimental design

Fermentation conditions (initial pH and temperature) of the yellow mombin juice by *L*. *acidophilus* were optimized using a 2^2^ central composite rotational design (CCRD) with 4 axial points and quadruplicate at the central point, totaling 12 assays. The initial pH adjustment of the juices was performed by adding 4 M sodium bicarbonate (Êxodo Científica, Sumaré, Brazil). Next, the 5 mL volume of the MRS broth activated strain was aseptically transferred to 250 mL Erlenmeyer flasks containing 100 mL of the juice under different pH conditions. The flasks were incubated for 24 h at different temperatures to assess viable cell count. The levels used in the CCRD are presented in Table 1.

**Table 1:** Composition of yellow mombin juice formulations used in sensory analysis.

Formulation	Composition
**F1**	Standard yellow mombin juice (Unfermented)
**F2**	Yellow mombin juice fermented with *L*. *acidophilus* for 16 h
**F3**	Yellow mombin juice fermented with *L*. *acidophilus* for 16 h and sweetened with 10% stevia powder

### Evaluation of fermentation time

Erlenmeyer flasks containing 100 mL of the yellow mombin juice were inoculated with *L*. *acidophilus*. Fermentation was carried out at the operating conditions of initial pH (6.4) and temperature (30°C), as previously determined by CCRD. The flasks were incubated for up to 24 h, with samples taken every 2 h for viable cell count. The remainder of the sample was centrifuged at 1200 xg (Model 80-2B, Centribio, São Paulo, Brazil) at room temperature for 15 min. Next, pH, titratable acidity, total soluble solids, total proteins, phenolic compounds and antioxidant activity were analyzed in the supernatant.

### Viable cell count

The viability of *L*. *acidophilus* in fermented yellow mombin juice was evaluated according to the methodology previously described [16]. Serial dilutions were made in 0.3% peptone water (HiMedia Laboratories, Mumbai, India) in 10^−1^ to 10^−10^ for each sample. Then 100 μL of dilutions 10^−5^ to 10^−10^ were seeded on plates containing MRS agar. The plates were incubated at 37°C for 72 h. Viability was expressed in logarithm of Colony Forming Units per mL (Log CFU/mL).

### Chemical composition, color, phenolic components and antioxidant activity of the yellow mombin juice

The pH was determined by direct measurement in a digital potentiometer. The measurement of total soluble solids—TSS (°Brix) was performed using a digital refractometer (Quimis, Q767BD, Diadema, Brazil) and the concentration of lactic acid and acetic acid was determined by high-performance liquid chromatography (HPLC, Shimadzu Corp., Kyoto, Japan) [25].

Total protein concentration was determined by the Bradford method [26] using bovine serum albumin (BSA) as standard (Sigma Aldrich Co., St. Louis, MO, USA). We assumed the BSA as being 100% pure. Color analysis was performed at 24 ± 1°C using an ACR 1023 color analyzer (Instrutherm Instrumentos de Medição Ltda, São Paulo, Brazil). The parameters were obtained in the RGB system (red, green and blue) and converted to the CIE Lab color space using the OpenRGB software and expressed in color coordinates (L*, a* and b*). Total phenolic compounds were analyzed by the Folin-Ciocalteau method, according to Fujita et al. [27]. Phenolic components were determined by HPLC according to the methodology described by Padilha et al. [28]. The antioxidant activity in the juice was evaluated by the DPPH radical sequestration method [29] and the ABTS radical sequestration method [30].

### Determination of antibacterial activity

Antibacterial activity in the fermented juice supernatant was evaluated by the method previously standardized by the Clinical and Laboratory Standards Institute [31]. The following four bacterial strains were tested: *Staphylococcus aureus* ATCC 29213, *Enterococcus faecalis* ATCC 4028, *Escherichia coli* ATCC 25922 and *Klebsiella pneumonie* ATCC 10031.

The microbial suspension at 1.0 x 10^8^ CFU/mL (5 μL) was added to 100 μL BHI broth (HiMedia Laboratories) and 100 μL juice supernatant (cell-free). The microplate was incubated for 24 h under shaking at 200 rpm at 37°C (Quimis, Q816M20, Diadema, Brazil). The absorbance reading was taken at a wavelength of 492 nm (DR200Bs microplate reader, Hiwell Diatek Instruments Co., Wuxi, China). The inhibition percentage of microbial growth was determined from the spectrophotometric reading according to Eq 1 [32]:

Inhibition%ofmicrobialgrowth=[1‐(Ac/A0)]x100 (1)

Where Ac represents the mean absorbance of the tested juice already subtracted from the absorbance value obtained for the juice without the addition of inoculum, and A0 the average absorbance of the microbial growth control (without the juice).

### Microbiological stability of fermented beverage for up to 28 days

The fermented yellow mombin juice obtained under optimized conditions was stored in sterile tubes, closed and stored in a refrigerator at 4ºC. *L*. *acidophilus* viability was determined before storage and at 7-day intervals over 28 days [33].

### Survival of *L*. *acidophilus* NRRL B-4495 under simulated gastrointestinal conditions

The survival evaluation of *L*. *acidophilus* NRRL B-4495 under simulated gastrointestinal conditions was performed before and after 28 days of juice storage under refrigeration according to the previously described methodology [23,34]. The assays were performed using sterile Falcon tubes of 15 mL. *L*. *acidophilus* cells were resuspended in 10 mL of 0.85% NaCl solution containing 3 g/L pepsin (Sigma-Aldrich) after pH adjustment to 2.0 by the addition of 5 M HCl (simulated gastric conditions—SGC). Samples were incubated at 37°C and 70 rpm shaking (Quimis, Q816M20), removing 1 mL aliquots at times 0, 60 and 120 min. The cells were then left in simulated intestinal fluid (Simulated Intestinal Conditions—SIC), obtained by adding 1 g/L pancreatin (Sigma-Aldrich) and 10 g/L bile salts (Sigma-Aldrich) in 0.85% NaCl solution and the pH was adjusted to 8.0 with 2.0 M NaOH solution. Samples were incubated at 37°C and 70 rpm shaking (Quimis, Q816M20), and removing 1 mL aliquots at 180 and 240 min.

### Sensorial analysis

A total of 88 non-trained volunteer tasters were selected (81.81% women and 18.18% men). The tasters were between 18 and 46 years old. The analysis was conducted in individual cabins under appropriate lighting and temperature conditions. Table 1 shows the compositions of the analyzed formulations.

The overall acceptance t-test of the probiotic juice was performed by a hedonic scale ranging from 1 to 9, with 1–4 being the rejection zone, 5 the indifference zone, and 6–9 the acceptance zone. Acceptance was also evaluated through a Just-About-Right (JAR) ideal scale test which evaluated acidity, sweet taste, viscosity and color attributes. This study was approved by the research ethics committee of UFRN (CAAE 07811118.9.0000.5292). All volunteers signed an informed consent form to participate in the study.

### Statistical analysis

All central composite rotational design experiments were performed randomly and the data were processed using Statistica 8.0 software (StatSoft Inc., Tulsa, USA). The statistical significance of the second-order model equation was determined by the F-test (ANOVA).

The juice characterization results were expressed as arithmetic mean (standard deviation). Statistical analysis was performed using one-way ANOVA with Tukey’s post-test, considering the 95% confidence level (p <0.05) as a significant result. Experiments and measurements were performed in duplicate.

The sensory analysis results were expressed as median, and the non-parametric Kruskal-Wallis test with Dunn test was performed for multiple comparisons, while the Bonferroni correction was employed for the statistical analysis using XLStat software (Addinsoft, Paris, France).

## Results and discussion

### Optimization of the initial pH and juice fermentation temperature conditions

The effects of temperature and initial pH of yellow mombin juice on *L*. *acidophilus* cell viability response were evaluated after 24 h of fermentation using the 2^2^ central composite rotational design (CCRD). The viability response varied from 5.06 to 8.02 Log CFU/mL (Table 2).

**Table 2:** 2^2^ CCRD matrix for optimizing the temperature and initial pH conditions of the yellow mombin juice fermentation using *L*. *acidophilus*.

Assay	Temperature (ºC)	pH	Viability^a^ (Log CFU/mL)
**1**	23.0 (-1)	4.00 (-1)	6.01 ± 0.17
**2**	37.0 (+1)	4.00 (-1)	6.10 ± 0.26
**3**	23.0 (-1)	7.00 (+1)	6.65 ± 0.05
**4**	37.0 (+1)	7.00 (+1)	6.84 ± 0.11
**5**	20.1 (-1.41)	5.50 (0)	6.11 ± 0.07
**6**	39.9 (+1.41)	5.50 (0)	6.19 ± 0.07
**7**	30.0 (0)	3.38 (-1.41)	5.06 ± 0.02
**8**	30.0 (0)	7.62 (+1.41)	7.18 ± 0.00
**9**^**b**^	30.0 (0)	5.50 (0)	8.02 ± 0.06
**10**^**b**^	30.0 (0)	5.50 (0)	6.79 ± 0.00
**11**^**b**^	30.0 (0)	5.50 (0)	6.98 ± 0.03
**12**^**b**^	30.0 (0)	5.50 (0)	6.93 ± 0.05

^a^Result expressed in mean ± SD.

^b^Central point

A statistical model was constructed from the adjusted regression coefficients obtained by the Statistica 8.0 software which correlates *L*. *acidophilus* cellular viability (Log CFU/mL) with the temperature and initial pH factors (Eq 2). The model was statistically significant at 95% confidence level, since the calculated F value (6.63) was higher than the listed F value (F_1.10_ = 4.96).

$$Viability\;\left(Log\;CFU/mL\right)=7.18+0.05X_{1}‐0.45X_{1}{}^{2}+0.55\;X_{2}‐0.47\;X_{2}{}^{2}+0.02\;X_{1}X_{2}$$ (2)

In which: X_1_ is the temperature and X_2_ the initial pH of the juice.

**Fig 1:**
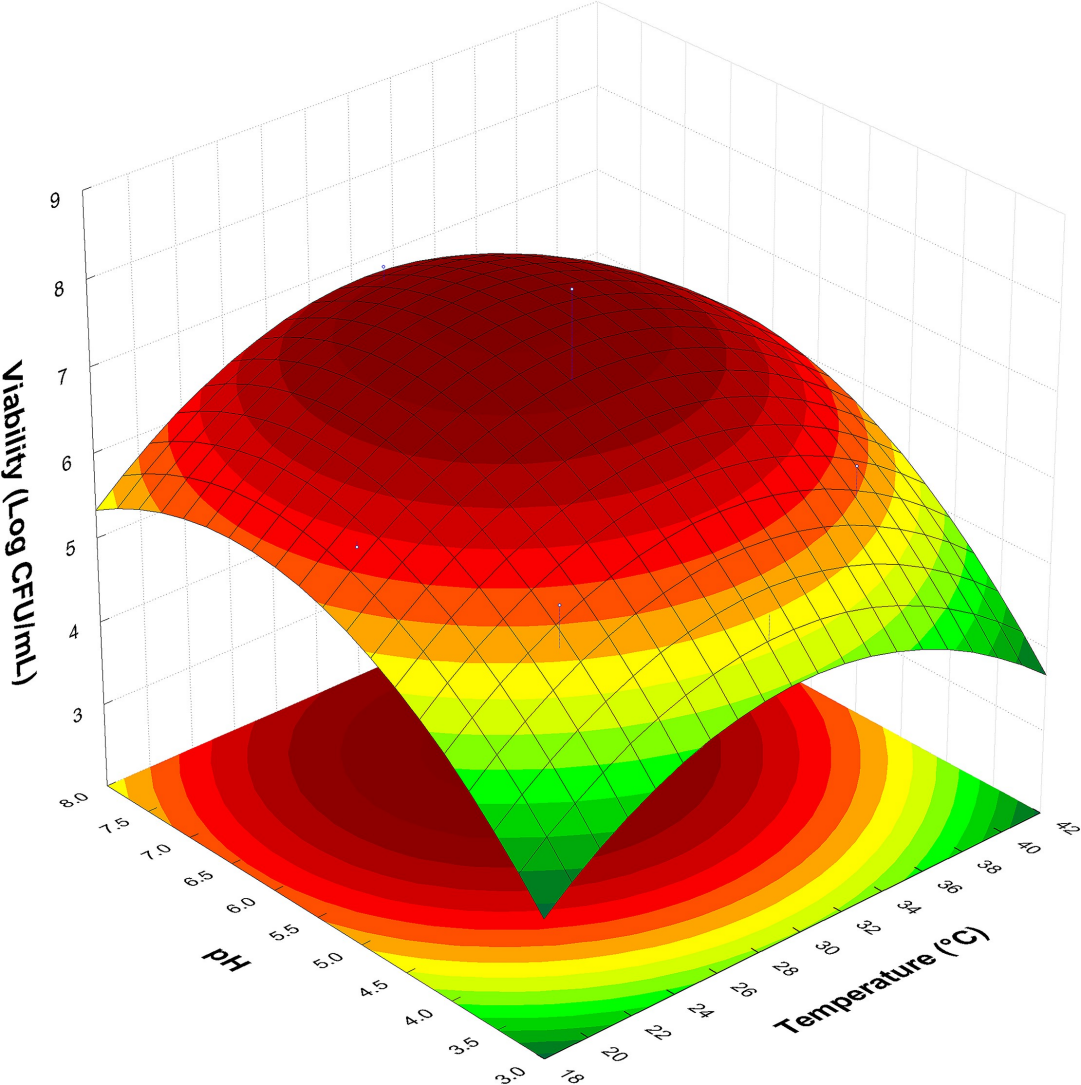
Response surface graph for cell viability of *L*. *acidophilus* (Log CFU/mL) fermented in yellow mombin juice for 24 h as a function of temperature and pH variables.

In observing the response surface graph (Fig 1), we can see that the critical point with higher cell viability in the present study was at pH 6.4. As a result, it was defined that the pH of the yellow mombin juice would be adjusted to 6.4 in all the following study steps.

Similar to the present study results, Santos Filho et al. [8] produced a probiotic beverage from *L*. *casei* NRRL-442 fermented cocoa juice, obtaining maximum viability at pH 6.2. Moreover, it was also observed that the highest growth and viability of *L*. *casei* NRRL-442 in cashew apple juice occurred at pH 6.4 [33].

As the statistical analysis revealed that the temperature variable did not influence the viability response of *L*. *acidophilus* in yellow mombin juice, the temperature for the next step was set at 30°C (central point). Yoon et al. [35] previously reported that *L*. *plantarum*, *L*. *casei* and *L*. *delbrueckii* grew rapidly on cabbage juice at 30°C. Pereira et al. [16] also described that *L*. *casei* grew well on cupuassu (*Theobroma grandiflorum*) beverage at 30°C.

### Evaluation of fermentation time

**Fig 2:**
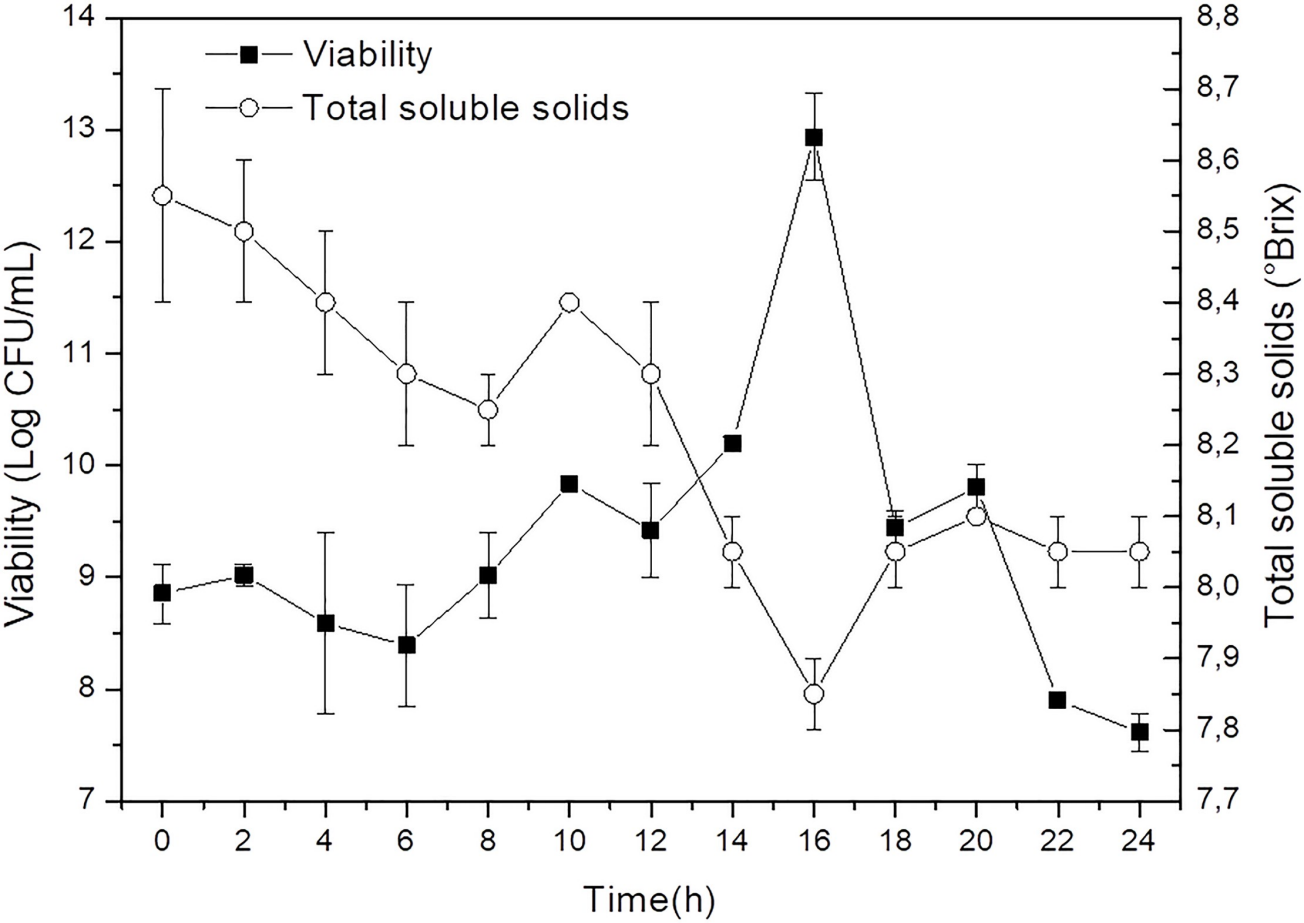
Cell viability profile of *L*. *acidophilus* (Log CFU/mL) and total soluble solids (°Brix) in yellow mombin juice fermented for 24 h.

The fermentation time was analyzed by counting viable cells for 24 h after determining the optimal pH and temperature conditions for *L*. *acidophilus* cell viability in yellow mombin juice (Fig 2).

According to Fig 2, it can be observed that there was an adaptation phase (lag phase) in the yellow mombin juice after inoculation. The microorganism showed exponential growth after 12 h of fermentation, reaching higher viability at 16 h with 12.9 ± 0.4 Log CFU/mL. As a result, this time was chosen as ideal for producing the probiotic yellow mombin juice.

It is important to highlight that the results presented in Fig 2 prove that *L*. *acidophilus* was able to ferment yellow mombin juice with satisfactory viability without adding sucrose or other nutrients. The decrease in TSS (Fig 2) shows that the juice sugars were partially used during fermentation. However, the increase in TSS in 18 h may be associated with the sucrose inversion process.

No significant variation in pH values was observed during the 24 h of fermentation, differing from other studies which report a substantial decrease in pH. This can be explained by the need to adjust the pH of the yellow mombin juice, which is naturally acidic (~2.0), and was changed with the addition of sodium bicarbonate until reaching the ideal pH of 6.4, as defined in the CCRD. Thus, a buffering effect may have been generated, constituting a hypothesis which would explain this ability to resist pH changes.

### Characterization of fermented juice

After determining the best fermentation conditions, fermented yellow mombin juice (FJ) and unfermented juice (UFJ) were evaluated for chemical composition, color, phenolic components and antioxidant activity. Table 3 shows the characterization of unfermented and fermented juice by *L*. *acidophilus* for 16 h.

**Table 3:** Result of chemical composition, color, phenolic components and antioxidant activity in unfermented and fermented juice.

Parameter	Yellow mombin juice^a^	
	Unfermented	Fermented
**Lactic acid (g/L)**	0.03 ± 0.00^**a**^	0.92 ± 0.09^b^
**Acetic acid (g/L)**	0.04 ± 0.00^**a**^	0.77 ± 0.13^b^
**Total protein (mg/mL)**	0.13 ± 0.00^**a**^	0.19 ± 0.00^b^
***Color***		
L*	21.98 ± 0.01^**a**^	13.72 ± 0.21^b^
a*	13.65 ± 0.05^**a**^	5.17 ± 0.90^b^
b*	22.64 ± 0.47^**a**^	10.66 ± 0.71^b^
**Total phenolics (μg GAE/mL)**	609.31 ± 8.83^**a**^	94.90 ± 7.12^b^
***Phenolic components***		
**Gallic acid (μg/mL)**	34.82 ± 2.09^**a**^	25.44 ± 0.99^b^
**Catechin (μg/mL)**	1.59 ± 0.11	ND
**Ellagic acid (μg/mL)**	1.27 ± 0.25	ND
**Quercetin (μg/mL)**	2.65 ± 0.70^**a**^	2.94 ± 0.53^**a**^
**Eugenol (μg/mL)**	1.00 ± 0.15^**a**^	0.38 ± 0.02^**a**^
***Antioxidant activity***		
**DPPH (μmol TE/mL)**	3.28 ± 0.02^**a**^	0.31 ± 0.00^b^
**ABTS (μmol TE/mL)**	3.56 ± 0.27^**a**^	2.59 ± 0.30^b^

Different letters on the same line indicate a statistical difference between the means at 95% confidence by the Tukey test (p<0.05).

^a^Result expressed in mean ± SD.

Although the pH did not significantly change during the fermentation, it was possible to observe a variation in the lactic acid and acetic acid concentration values in the 16 h of fermentation (Table 3). The addition of sodium bicarbonate to reach the ideal fermentation pH (6.4) generated a buffering effect in the juice. Thus, lactic and acetic acids produced during fermentation were not able to reduce the pH. The unprotonated forms (acetate and lactate) predominate at the evaluated pH.

Despite the low protein concentration found in the juice, Sameh et al. [36] describe that the *Spondias* genus species are rich in free amino acids such as glycine, cysteine, serine, alanine and leucine. Therefore, the presence of these free amino acids in the yellow mombin juice represents an additional advantage, and could also justify the good adaptation of *L*. *acidophilus* in the studied matrix.

It is noticed that the luminosity parameter (L*) values decreased after fermentation, making the juice darker. In addition, the a* and b* coordinates significantly differed between UFJ and FJ, confirming that the *L*. *acidophilus* fermentation in juice interfered with these color parameters. In a similar way to that observed in our study, Costa et al. [37] reported that the addition of *L*. *paracasei* promoted browning in orange juice, with a consequent loss of yellow color.

The phenolic concentration in the UFJ was almost five times higher than that found in the FJ. It is suggested that the pH adjustment of the yellow mombin juice carried out before fermentation is responsible for the drop in the values of phenolic compounds in the FJ. As seen in Table 3, the gallic acid, catechin and ellagic acid concentrations were significantly reduced (p<0.05) in the fermented product.

The sample’s antioxidant activity without pH adjustment was also significantly higher as a result of the higher phenolic compounds content in the UFJ. However, despite the decrease in FJ’s phenolic compounds and antioxidant activity, the probiotic presence is a differential and of great advantage compared to UFJ. Similarly to this study, Hashemi et al. [5] described a significant reduction in the total phenolic concentration in sweet lemon juice fermented with *L*. *plantarum* LS5, attributed to the metabolism of these components by the microorganism.

### Antibacterial activity

The results found in the present study show (Table 4) that there was growth inhibition of *Staphylococcus aureus* (44.5%) and *Escherichia coli* (35.0%), and less inhibition for *Klebsiella pneumoniae* (16.1%). A justification for the activity found is the production of compounds such as bacteriocins, lactic acid and hydrogen peroxide by *L*. *acidophillus*. Tejero-Sariñena et al. [38] described the significant antibacterial activity of *L*. *acidophillus* NCIMB 30184 against *E*. *coli* and *S*. *aureus* and *E*. *fecalis*, thus diverging from the result found in the present study since no inhibitory effect was detected against this microorganism.

**Table 4:** Antibacterial activity of the supernatant of yellow mombin juice fermented by *L*. *acidophilus* for 16 h.

Microorganism	Growth inhibition (%)ª
***Staphylococcus aureus***	44.5 ± 12.4
***Enterococcus faecalis***	ND^b^
***Escherichia coli***	35.0 ± 3.3
***Klebsiella pneumoniae***	16.1 ± 1.0

^a^Result expressed in mean ± SD.

^b^Not detected.

Probiotics, such as *L*. *acidophilus*, play a crucial role in maintaining the human gastrointestinal tract microbial ecosystem by the growth inhibition of ingested pathogens, offering alternatives to antibiotics for gastrointestinal infections [39]. In addition, the use of probiotics is a useful strategy for obtaining products with a longer shelf life and safer products due to their ability to slow or prevent the growth of contaminating bacteria [5].

### Microbiological stability of fermented beverage for up to 28 days

**Fig 3:**
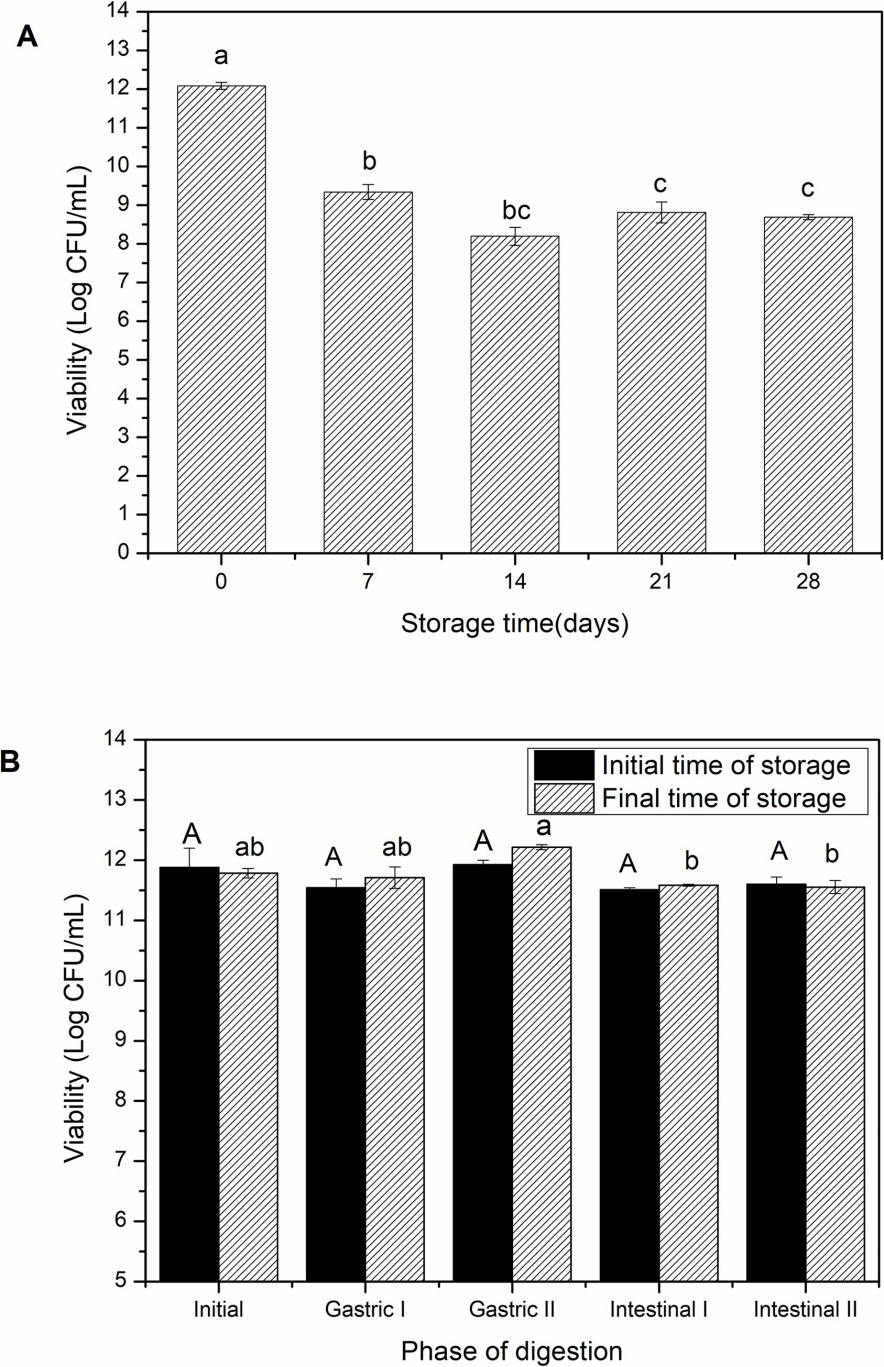
Microbiological stability of the fermented beverage during storage for 28 days (A), and cell viability of *L*. *acidophilus* NRRL B-4495 under simulated gastrointestinal conditions at the beginning and end of the 28-day refrigerated storage period (B). Equal letters indicate that there is no statistical difference between the 95% confidence means (p<0.05).

One of the major challenges in developing probiotic vegetable drinks is to ensure that the microorganism maintains its viability and functionality throughout the product’s shelf life. The microbiological stability of yellow mombin juice fermented with *L*. *acidophillus* and stored under refrigeration (4°C) was evaluated for 28 days in this study. The results are presented in Fig 3A.

The results showed that the viable cell count in the yellow mombin juice dropped from 12.0 ± 0.1 Log CFU/mL on day 0 to 9.3 ± 0.2 Log CFU/mL on the 7^th^ day, with the storage time significantly influencing the viability of *L*. *acidophillus* in the first 7 days (p<0.05). Its viability remained stable soon after on days 14, 21 and 28, with a count of 8.7 ± 0.7 Log CFU/mL at the end of the period. It is important to note that the count remained above 6.0 CFU/mL during the entire storage period, which corresponds to an amount greater than 10^6^ CFU/mL. Several authors claim that a probiotic product must have at least 10^6^ viable bacteria per gram of product over the entire shelf life to show its beneficial health effects [11,13,40].

### Survival of *L*. *acidophilus* NRRL B-4495 under simulated gastrointestinal conditions

The viability of *L*. *acidophillus* was analyzed *in vitro* to evaluate the survival of the microorganism in simulated gastrointestinal conditions and its suitability for the matrix by using the freshly fermented juice at the end of the storage time under refrigeration of 28 days.

It was found that the viability of the culture for the freshly fermented juice before exposure to gastric stress did not differ (p<0.05) from the viability of the gastric and intestinal phases when evaluating the probiotic behavior in the different phases. However, the juice refrigerated for 28 days showed significantly lower (p<0.05) viable cell counts for enteric phases I and II, dropping from 11.78 ± 0.03 Log CFU/mL at the beginning to 11.58 ± 0.00 Log CFU/mL and to 11.55 ± 0.05 Log CFU/mL, respectively (Fig 3B).

Despite the statistically significant reduction of the refrigerated probiotic in the simulated intestinal conditions for the drink stored for 28 days, it is essential to highlight that the viable cell count remained above 6.0 Log CFU/mL to provide the beneficial effects.

### Sensorial analysis

The acceptance of the yellow mombin probiotic beverage and the purchase intention was determined by a hedonic scale. Table 5 shows the acceptance index of the three formulations. The results showed that F1 was the best-accepted formulation (64.7%). Even so, this acceptance was considered low (<70.0%). On the other hand, F2 was had the lowest acceptance rate (36.5%). The lower acceptance percentages found for fermented formulations may be linked to the fact that fermentation changes the sensory characteristics common to juice through metabolite production. In addition, adjusting the pH of the yellow mombin juice (~2.0) to the ideal fermentation pH (6.4) causes changes in color (gets darker), viscosity (more viscous) and changes the flavor by adding sodium bicarbonate.

**Table 5:** Mean, median and acceptance index (AI) of each analyzed formulation.

Acceptance	F1	F2	F3
**Mean**	5.78 ± 1.97	2.19 ± 1.40	3.74 ± 2.25
**Median**	6ª	2^c^	3^b^
**AI (%)**	64.77	36.55	41.54

Different letters in the same line show that the medians of the formulations differed by the Kruskal-Wallis Dunn test and Bonferroni correction (p<0.05).

Despite F3 being a fermented sample under the same conditions as F2, it had a higher AI. As the sample was sweetened (stevia), it can be concluded that the sweet taste in this formulation pleased the tasters more in comparison with the fermented formulation without the addition of sweetener.

**Fig 4:**
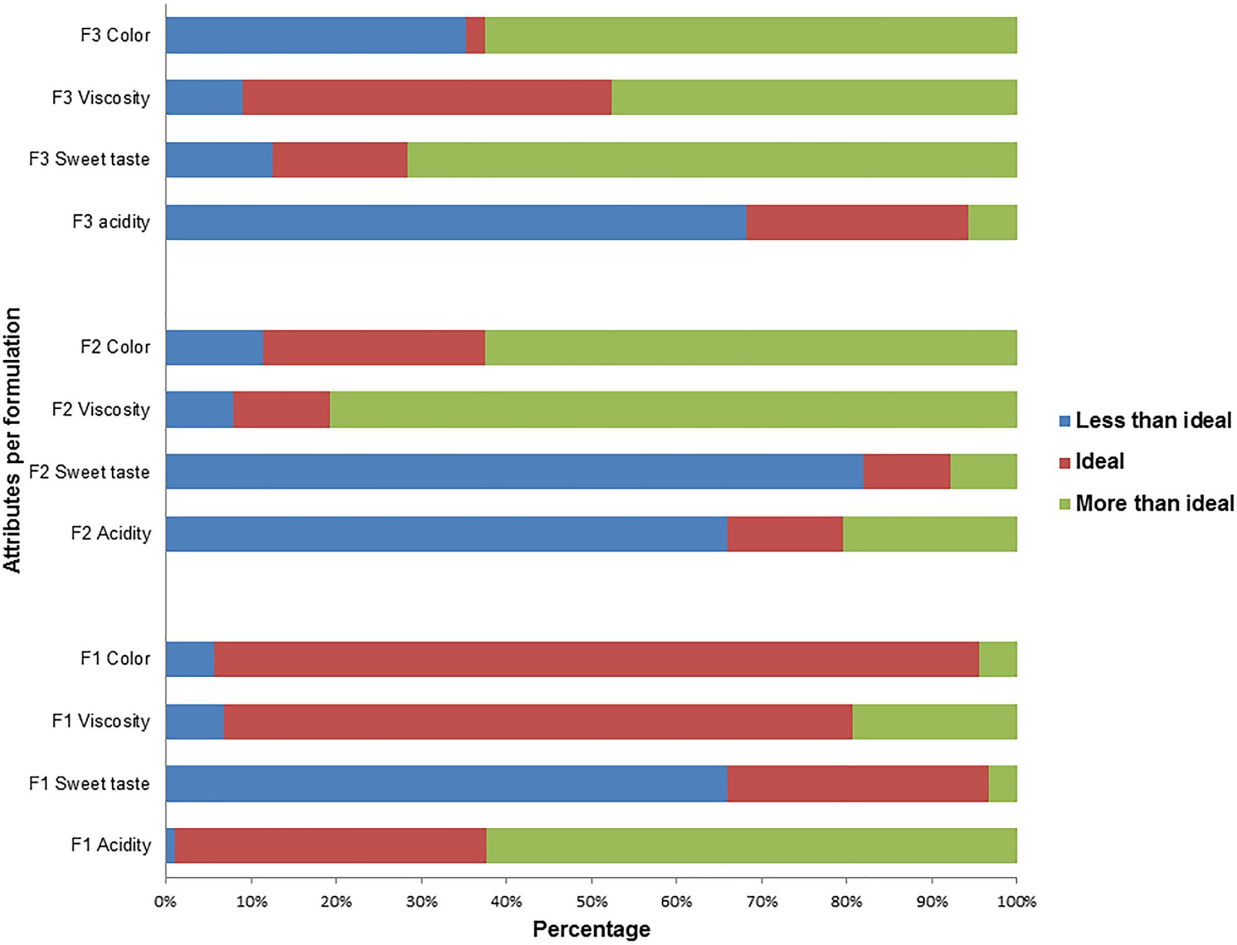
The influence intensity of the acidity, sweet taste, viscosity and color attributes in accepting the F1, F2 and F3 formulations.

The Just-About-Right (JAR) ideal scale test was performed to assess the intensity of the attributes acidity, sweet taste, viscosity and color in the acceptance of each formulation tested, as shown in Fig 4.

It is observed that the tasters chose the F1 sample as ideal among the formulations for the viscosity and color attributes. The results regarding the sweet taste and acidity attributes show that most of the tasters chose the extremes of the scale to classify the intensity of these attributes, with acidity being chosen by them as “more than ideal” (62.5%), and sweet flavor as “less than ideal” (65.9%).

On the other hand, in F2 we can observe that acidity and sweet taste are classified on the “less than ideal” scale (65.9% and 81.81%, respectively), while the viscosity and color attributes are on the “more than the ideal” end (80.7% and 62.5%, respectively). This demonstrates that the evaluators considered all the attributes of this formulation to be unsatisfactory. It is also seen that all attributes are considered outside of ideal for the F3 formulation. The acidity attribute was defined as "less than ideal" (68.2%), and the sweet flavor was also classified as "more than ideal" (71.6%). They also defined viscosity and color as “more than ideal” (47.7% and 67.5%, respectively).

Fermentation was responsible for altering some important sensory aspects, mainly the color and viscosity, which were very different in the fermented samples from the juice standard, and directly affected the taster’s acceptance. The penalty test was performed when more than 20% of the tasters chose an extreme to evaluate an attribute to confirm the influence of attributes on the taster’s acceptance of each formulation. The Supporting Information (S1, S2 and S3 Tables) presents the penalties obtained for each formulation.

Finally, another aspect of fundamental importance is that consumers need to be aware of the health benefits when functional ingredients such as probiotics are added to foods, and realize the product’s difference from conventional products. It is noteworthy that this is one of the most critical aspects of the acceptance of functional foods. Thus, the food industry must clearly and understandably communicate the beneficial health effects to consumers.

## Conclusion

From the results found in this study, we can conclude that the yellow mombin juice is a viable matrix for the growth of *L*. *acidophillus*, enabling viable cell counts to be maintained for 28 days of storage under refrigeration as good resistance against gastrointestinal stress. We can emphasize that the fermentation maintained the concentration of some phenolic compounds in the beverage and part of its antioxidant activity, giving the product a functional aspect. The fermented juice showed antibacterial activity against *S*. *aureus*, *E*. *coli* and *K*. *pneumoniae*. Although the acceptance regarding the sensorial analysis was not satisfactory, it was possible to establish the characteristics which did not please the tasters in the tested formulations, and thereby to establish the criteria which need improvements to make the formulations according to the consumers’ tastes. Even so, more studies are needed to improve the knowledge acquired to date. However, overall this study showed great potential for creating a new probiotic beverage based on yellow mombin juice, constituting an excellent alternative to traditional dairy products.

## Supporting information

S1 Table Penalty analysis for formulation F1 in percentage (%), effects on mean, and penalties (p-value). (PDF)

S2 Table Penalty analysis for formulation F2 in percentage (%), effects on mean, and penalties (p-value). (PDF)

S3 Table Penalty analysis for formulation F3 in percentage (%), effects on mean, and penalties (p-value). (PDF)

S1 Dataset (XLSX)
