# Supplementary material for: Fermented yellow mombin juice using Lactobacillus acidophilus NRRL B-4495: Chemical composition, bioactive properties and survival in simulated gastrointestinal conditions
Source: PLoS One. 2020 Sep 24;15(9):e0239392. doi: 10.1371/journal.pone.0239392 (PMC7514086; doi:10.1371/journal.pone.0239392)
Supplement: S1 Table — (PDF) [file pone.0239392.s001.pdf]

Supporting Information S1

Table S1. Penalty analysis for formulation F1 in percentage (%), effects on mean, and penalties (p-value).

Variable	Level	%	Effects on mean	p-value	Penalty	p-value
**Acidity**	Less acid	1.14	4.125	< 0.0001	2.107	< 0.0001
	Ideal	36.36				
	More acid	62.50	2.070			
**Sweet taste**	Less intense	65.91	2.289	< 0.0001	2.395	< 0.0001
	Ideal	30.68				
	More intense	3.41	4.444			
**Viscosity**	Less viscous	6.82	1.864		0.944	0.048
	Ideal	73.86				
	More viscous	19.32	0.619			
**Color**	Very clear	5.68	0.048		0.626	0.370
	Ideal	89.77				
	Darker	4.55	1.348			
