# Supplementary material for: Fermented yellow mombin juice using Lactobacillus acidophilus NRRL B-4495: Chemical composition, bioactive properties and survival in simulated gastrointestinal conditions
Source: PLoS One. 2020 Sep 24;15(9):e0239392. doi: 10.1371/journal.pone.0239392 (PMC7514086; doi:10.1371/journal.pone.0239392)
Supplement: S2 Table — (PDF) [file pone.0239392.s002.pdf]

Supporting Information S2

Table S2. Penalty analysis for formulation F2 in percentage (%), effects on mean, and penalties (p-value).

Variable	Level	%	Effects on mean	p-value	Penalty	p-value
**Acidity**	Less acid	65.91	1.236	0.014	1.127	0.009
	Ideal	13.64				
	More acid	20.45	0.778	0.277		
**Sweet taste**	Less intense	81.82	0.319	0.531	0.404	0.417
	Ideal	10.23				
	More intense	7.95	1.270			
**Viscosity**	Less viscous	6.82	-0.091		0.818	0.071
	Ideal	12.50				
	More viscous	80.68	0.895	0.046		
**Color**	Very clear	4.55	0.848		0.209	0.542
	Ideal	26.14				
	Darker	69.32	0.167	0.634		
