# Supplementary material for: Fermented yellow mombin juice using Lactobacillus acidophilus NRRL B-4495: Chemical composition, bioactive properties and survival in simulated gastrointestinal conditions
Source: PLoS One. 2020 Sep 24;15(9):e0239392. doi: 10.1371/journal.pone.0239392 (PMC7514086; doi:10.1371/journal.pone.0239392)
Supplement: S3 Table — (PDF) [file pone.0239392.s003.pdf]

Supporting Information S3

Table S3. Penalty analysis for formulation F3 in percentage (%), effects on mean, and penalties (p-value).

Variable	Level	%	Effects on mean	p-value	Penalty	p-value
Acidity	Less acid	68.18	2.515	<0.0001	2.473	< 0.0001
	Ideal	26.14				
	More acid	5.68	1.965			
Sweet taste	Less intense	12.50	2.136		3.284	< 0.0001
	Ideal	15.91				
	More intense	71.59	3.484	< 0.0001		
Viscosity	Less viscous	9.09	1.184		1.664	0.000
	Ideal	43.18				
	More viscous	47.73	1.756	0.000		
Color	Very clear	2.27	-0.597		1.798	0.000
	Ideal	35.23				
	Darker	62.50	1.885	0.000		
